# Environmental Impacts of Engineered Nanomaterials—Imbalances in the Safety Assessment of Selected Nanomaterials

**DOI:** 10.3390/ma11081444

**Published:** 2018-08-15

**Authors:** Dana Kühnel, Harald F. Krug, Anita Jemec Kokalj

**Affiliations:** 1 Department Bioanalytical Ecotoxicology (BIOTOX), Helmholtz Centre for Environmental Research, GmbH—UFZ, Permoserstraße 15, 04318 Leipzig, Germany; 2 NanoCASE GmbH, St. Gallerstr. 58, 9032 Engelburg, Switzerland; hfk@nanocase.ch; 3 Biotechnical Faculty, University of Ljubljana, Jamnikarjeva 101, 1000 Ljubljana, Slovenia; anita.jemec@bf.uni-lj.si

**Keywords:** engineered nanomaterials (ENMs), environmental release, environmental fate, ecotoxicity, nanosafety, DaNa project

For this Editorial, we, the guest editors, performed a brief review with the aim of setting the framework for the Special issue on the “Environmental Impacts of Nanomaterials”. We started with the DaNa web-based knowledge base (accessible through www.nanoobjects.info) and assessed the number of studies conducted for each engineered nanomaterial (ENM) represented in the knowledge base. Specifically, studies with published data in the fields of environmental release, toxicity and ecotoxicity of ENMs were considered. 

In general, engineered nanomaterials (ENM) are a diverse group of materials that find application in a great variety of products ranging from cosmetics to paints, from sports equipment to electronics, and from soil remediation to nanomedicine [1]. The Nanotechnology Consumer Products Inventory lists 1827 products containing ENMs [2]. Depending on the type of product and the integration of the nanomaterial into it, the release of engineered nanomaterials into the environment is inevitable [3].

The environmental impact of ENMs is a broad field spanning not only the whole life cycle of nanomaterials, including the waste phase, but also many different environmental contexts with different prevailing conditions. Ultimately, ENM will also reach the technosphere, for example, waste water treatment or incineration plants. In addition to incidental release, there are several applications that imply the intentional release of ENM into water and soil, such as ENM employed in remediation techniques [4]. 

Considering the increasing use and application of ENMs, there has been growing concern about the environmental safety and impact of nanomaterials released to various environmental compartments. During the last decade tremendous effort has been put into scientifically addressing these concerns, resulting in an overwhelming number of studies. In 2009, the DaNa project was launched to summarize and aggregate the knowledge generated and aimed to provide impartial information on ENM with access for all. For this purpose, knowledge from the scientific literature, as well as other sources is condensed into a web-based knowledge base (for more information, see www.nanoobjects.info [5,6,7,8]. Currently, the knowledge base covers 26 different types of ENM, ranging from metals and metal oxides to organic materials such as cellulose. Another goal of this database is to gather information on the release, transport, fate and effects of ENM in the environment. From our extensive literature search, however, it is evident that the coverage varies with regard to available information on the 26 nanomaterials. While numerous studies have been conducted for nanoscaled silver, titanium dioxide and zinc oxide, little to no information exists for nanomaterials such as indium tin oxide (ITO), barium sulfate or strontium carbonate. In general, there are many laboratory ecotoxicity studies involving a range of test organisms (mostly aquatic ones) [9,10], but there is much less information available on the release and exposure, and the behavior of ENMs under environmental conditions (transport and transformation). 

In order to provide an overview of these issues, an ISI-WoS (Thomson Reuters Web of Science) was conducted for the 26 ENMs included in the DaNa knowledge base. The search strategy used the name of the respective ENM, “ENM” and “nano*” and either “environment”, “tox*”, or “ecotox*” as keywords. The results are shown in Table 1. As evident from the collection of studies that were retrieved for each given nanomaterial, most research has focused on the synthesis and general development of a nanomaterial for a specific application. With regard to nano-safety, as indicated by the number of studies found for the general search term “tox*”, most research has been done for nanogold, nanoiron, nanosilver and quantum dots. These ENMs have important medical applications such as cancer therapeutics, contrast agents, diagnostics and vaccination. Also substantial research has been conducted for nanoscaled zeolites, diamond, aluminium oxide, iron and iron oxide, and titanium dioxide, which have promising applications in the environmental sector, or as in the case of titanium dioxide, its application in sunscreens implies potentially high release into the environment,. This is demonstrated by the number of studies retrieved by applying the additional search term “environment”. Also, research related to environmental hazard (search term “ecotox*”) is evident, except for issues related to nanodiamond and nanoaluminium oxide which were addressed in only a few studies. Interestingly, the most researched nanomaterials with regard to environmental safety include nanosilver, nanozinc and nanocopper, probably because they are used in many applications due to their antiseptic actions, which also implies the release of ions into the environment.

This rather simple evaluation of current nanotechnology related literature reveals discrepancies in the assessment of the safety of selected nanomaterials. In particular, current research on environmental safety tends to focus on whether applications have relevance in the environmental sector rather than assessment of actual release and distribution in different environmental settings. With regard to the latter, the literature search applying the additional term “environmental release” yielded either none or very few results. Most of the currently available data on this issue relies on modelling approaches e.g., [11,12].

In addition, many of the research gaps evident in the field of nanotechnology are due to insufficient, very time consuming and/or expensive methodology and analytical procedures. Detection and quantification of ENMs in complex environmental samples are particularly challenging [13,14].

As evidenced by the number of hits given in Table 1 for the keywords “ENM”, “nano*” and “tox*”, and “material”, “nano*” and “ecotox*”, a multitude of studies are available for some nanomaterials. The DaNa project team tries to evaluate this body of literature, in particular, the reliability of these studies. Toxicity studies involving nanomaterials require specifically adopted test procedures and need to consider a number of particle-specific issues due to the unique properties and behavior of ENMs [15,16]. Still, not all studies use appropriate methodology when testing ENMs, leading to inaccurate or irreproducible results for these nano(eco)toxicity studies. In order to provide a reliable foundation for the DaNa knowledge base, the DaNa project team developed the DaNa criteria checklist [17] to evaluate the quality of studies related to nanosafety and select appropriate studies. Additionally, the checklist may also be used to prepare and design studies in the field of nano(eco)toxicity and it is also feasible for evaluating the validity of no-effect studies. In these types of studies, judging whether the absence of biological effects is due to non-toxicity of the test item or to experimental error is particularly challenging. For example, this may involve using ENM concentrations close to realistic or predicted environmental concentrations. 

We hope that our brief literature review provides some inspiration to steer research into the impact of ENMs on the environment in a more appropriate direction. As discussed, there are specific data gaps in the area of environmental risk assessment. In order to improve the evaluation of the environmental risks of ENMs, we recommend generating more data of high quality and reliability on the actual environmental release and fate of ENMs. Furthermore, we consider it important to foster the publication of ecotoxicity studies that report on the non-toxicity of ENMs as reliable studies on this issue represent important building blocks for improved risk assessment. 

Finally, to add to the ever-growing knowledge base in general, and subsequently to the DaNa knowledge base, in this special issue we aimed at providing a platform for more detailed research into the environmental effects of ENM. We, the guest editor team, hope that this Special Issue of Materials is of interest to the scientific community and facilitates further discussion on the environmental fate and safety of ENMs. 

## Figures and Tables

**Table 1:** Overview of the 26 different ENMs included in the DaNa knowledge base. The number of publications as retrieved in ISI Thomson Reuters Web of Science using the keywords “material” and “nano*” and either “environment”, “tox*” or “ecotox*” are shown. if those search terms did not deliver meaningful results, more specific ones were used as indicated in the respective box (accessed 17 November 2017). The number of publications is informative, because the publications were not filtered any further and may contain accidental hits. ENM in *italics* mark those with known applications according to the DaNa knowledge base (https://www.nanopartikel.info/en/nanoinfo/knowledge-base) in the environmental sector as specified in brackets. Materials are listed alphabetically.

Nanomaterial in DaNa Knowledge Base (Application in the Environmental Sector)	No. of Hits Keywords “ENM” According to First Column and “Nano*”	No. of Hits Keywords “Material” and “Nano*” and “Tox*”	No. of Hits Keywords “Material” and “Nano*” and “Environment”	No. of Hits Keywords “Material” and “Nano*” and “Ecotox*”
*Aluminium Oxides (Filtration)*	*1955*	*59*	*78*	*2*
Barium Sulphate	326	11	4	-
Carbon Black	8344	433	290	18
Carbon Nanotubes “CNT”	20,277	601	708	27
Cellulose	16,752	527	599	13
Cerium dioxide	857	179	78	25
Copper Copper Oxide	43,484	1817	1531	114
	10,641	776	505	71
*Diamond “nanodiamond” (Water Treatment)*	(16,381 *) 2600	*103*	*116*	*1*
Fullerenes	9899	703	580	95
Graphene	86,181	2397	2606	37
*Gold (Water Treatment)*	*109,533*	*5211*	*4446*	*89*
Indium Tin Oxide (ITO)	6301	56	145	4
*Iron* *Iron Oxide* *(Remediation, Water Treatment)*	*53,280*	*3412*	*2783*	*98*
	*28,621*	*2534*	*1558*	*51*
Nanoclays	3474	60	98	2
Platinum	26,173	534	804	7
“Quantum Dots”	100,562	3666	3160	69
Silicon Dioxide	3862	138	130	9
Silver	65,043	5858	3854	401
Strontium Carbonate	176	5	12	-
*Titanium Dioxide (Water Treatment)*	*23,953*	*2738*	*3107*	*280*
Titanium Nitride	2323	14	105	1
Tungsten Carbide	1804	31	69	-
Tungsten Carbide Cobalt	217	20	11	-
*Zeolites (Filtration, Fertilizer, Water Treatment)*	*10,142*	*183*	*417*	*2*
Zinc Oxide	23,577	1802	942	113
Zirconium Dioxide	582	25	14	1

* biased, because in many studies dealing with CNT, C_60_ or graphene, diamond is mentioned as another form of carbon-based ENM.
